# Recombined humanized endostatin (Endostar) intravenous infusion in the treatment of refractory nasopharyngeal carcinoma: Three case reports

**DOI:** 10.1097/MD.0000000000016592

**Published:** 2019-08-09

**Authors:** Chen Chen, Song-Ran Liu, Shu Zhou, Xiao-Hui Li, Xiao-Hui Wang, Ya-Lan Tao, Hui Chang, Wen-Wen Zhang, Wen-Fei Li, Si-Lang Zhou, Yun-Fei Xia

**Affiliations:** Department of Radiation Oncology, Cancer Center, Sun Yat-sen University, Guangzhou, Guangdong, China.

**Keywords:** antiangiogenic agent, combined therapy, endostar, objective response rate, refractory nasopharyngeal carcinoma

## Abstract

**Rationale:**

Refractory nasopharyngeal carcinoma is challenging to treat and at present there is no standard treatment or any good choice.

**Patient concerns:**

Although the three patients in our case reports had already underwent multiple treatments before, they still suffered from disease recurrence of nasopharyngeal carcinoma.

**Diagnosis:**

They were diagnosed as refractory nasopharyngeal carcinoma.

**Interventions:**

A continuous infusion of Endostar, an antiangiogenic agent, combined with chemotherapy and radiation therapy was given to treat the patients.

**Outcomes:**

Patients showed complete or partial response to the combined therapy as evidenced by regression of tumors and decrease in plasma Epstein–Barr virus (EBV) DNA load.

**Lessons:**

Continuous infusions of Endostar in combination with chemotherapy and/or radiation therapy showed promising efficacy and safety. The combination therapy indicates a new approach to treat refractory nasopharyngeal carcinoma.

## 1 Introduction

Nasopharyngeal carcinoma (NPC) is a type of head and neck cancer that is commonly found in Southeast Asia and evidence shows that Epstein–Barr virus (EBV) is highly associated with NPC.^[1]^ With the development of novel treatments, including chemotherapy and radiotherapy, the 5-year overall survival rate of patients with NPC is higher than 80%.^[2,3]^ However, one of the key issues in the treatment of NPC is recurrence, which may occur with radiation brain injury after radiotherapy, after the second or more courses of radiotherapy, and after failure of first-line treatment with multiple distant metastasis (either synchronous or metachronous). The characteristics of these patients are that they usually have complex treatments, are at a high risk of relapse, and have poor prognoses.^[4,5]^ These characteristics define refractory nasopharyngeal carcinoma. There is no standard treatment for refractory nasopharyngeal carcinoma. Platinum plus fluorouracil (5-Fu) chemotherapy is still the commonly used regimen.^[6]^

Given the fact that tumor growth and metastasis depend on angiogenesis,^[7]^ the effects of antiangiogenic agents as a treatment option have been explored in preclinical studies and clinical trials. Endostatin is an antiangiogenesis agent that is found in human circulation^[8,9]^ and was shown to be able to suppress tumor growth.^[10]^ Endostar is a recombinant human endostatin that has a strong antiangiogenesis effect; furthermore, preclinical and clinical studies have demonstrated its effectiveness in treating various types of cancers.^[11–13]^ Therefore, Endostar as a possible treatment for refractory NPC has been explored in NPC mice models and clinical trials.^[13–15]^ In our clinical practice, Endostar also achieved remarkable effectiveness in refractory NPC treatment. Based on this clinical evidence, we designed a clinical trial named *A Phase II Clinical Trial of Chemotherapy With or Without Endostar Continuous Intravenous Infusion in Refractory NPC* (registered on ClinicalTrials.gov: NCT02590133), which was in progress to assess its efficacy and safety as a new treatment for refractory NPC.

## 2 Methods

We identified 3 typical cases of refractory NPC and proposed a novel treatment of continuous infusion of Endostar in combination with chemotherapy and radiation therapy for these patients. The treatment is nedaplatin plus continuous low dose 5-Fu intravenous infusion combined with Endostar (Recombinant Human Endostatin Injection) continuous intravenous infusion, and concurrent low dose radiation therapy to the distant metastasis organ or sites.

The chemotherapy cycle was 60 days, and the dose was designed as: Endostar, 15 mg/m^2^/d, continuous intravenous infusion (2 mL/h) for 30 days each cycle; 5-Fu, 200 mg/m^2^/d, continuous intravenous infusion (2 mL/h) for 30 days each cycle; Nedaplatin, 80 mg/m^2^/d (IV drip) on d1 and d28 each cycle. Low dose radiotherapy was given to the whole organ of metastasis site located at 100 to 120 cGy per fraction (for vertebral metastasis, the whole organ defined as from the upper one to the lower one vertebra), while traditional dosage was given to the metastasis sites. Radiotherapy was combined at the beginning of the first cycle of chemotherapy, and at the third cycle if complete remission was not achieved after 2 cycles of chemotherapy.

Tumor responses were assessed by positron emission tomography-computed tomography (PET/CT) or CT or magnetic resonance imaging (MRI) depending on tumor site and were evaluated by response evaluation criteria in solid tumors (RECIST) 1.1. Adverse effects were assessed by common terminology criteria for adverse events (CTCAE) V5.0 grading. All the patients have provided written informed consent for publication of the cases.

## 3 Case descriptions

### 3.1 Case 1

**Figure 1:**
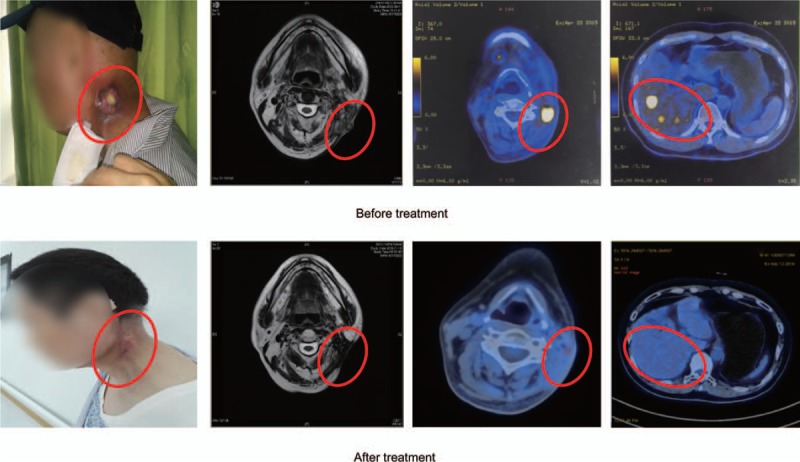
Imaging evaluation of treatment response for patient 1. Before treatment (April 2015), tumor was found to be progressive with recurrence in the lymph nodes in the neck and metastasis in the liver at multiple sites. After treatment (September 2016), oblong patchy shadows were observed in levels II and III of the neck, but no obvious abnormal structures or radioactive distributions in the liver were observed.

A 44-year-old Chinese man was initially diagnosed with stage III (T3N1M0) NPC with an EBV DNA load of 9.8 × 10^2^ copies/mL in August 2013 in our center. He was treated with concurrent 2 cycles of cisplatin (DDP) (75 mg/m^2^, once every 3 weeks) plus intensity-modulated radiation therapy (IMRT) in step and shoot delivery mode. At a follow-up visit in June 2014, tumor recurrence was found in the level II lymph nodes in the left neck on a PET/CT scan, and EBV DNA load increased to 1.14 × 10^5^ copy/mL in November 2014, the patient was treated with 4 cycles of lobaplatin plus 5-Fu with concurrent radiation therapy (40 Gy/20 Fr) to the recurrent lymph nodes in the left neck and 2 cycles of cytokine-induced killer cells (CIK) cytoimmunotherapy. However, a PET/CT scan obtained in April 2015 revealed the disease to be progressive with recurrence in the lymph nodes in the neck and metastasis in the liver at multiple sites (Fig. 1, before treatment). From May to August 2015, the patient was treated with 4 cycles of paclitaxel combined with cisplatin plus C225. A subsequent MRI examination found no significant changes in the size of the tumors either in the liver or the left neck. Plasma EBV DNA load of the patient was 1.92 × 10^6^ copies/mL.

From September 2015 to July 2016, the patient underwent 6 cycles of our proposed therapy as described in method with concurrent radiation therapy (24 Gy/12 F) for liver metastases. The patient's plasma EBV load was undetectable by August 2016. Oblong patchy shadows were observed in levels II and III of the neck, but no obvious abnormal structures or radioactive distributions in the liver were observed on the PET/CT scan obtained in September 2016 (Fig. 1, after treatment). Tumor response was evaluated as complete response (CR) by RECIST 1.1 and hematologic toxicity of the treatment is acceptable (Table 1).

**Table 1:**
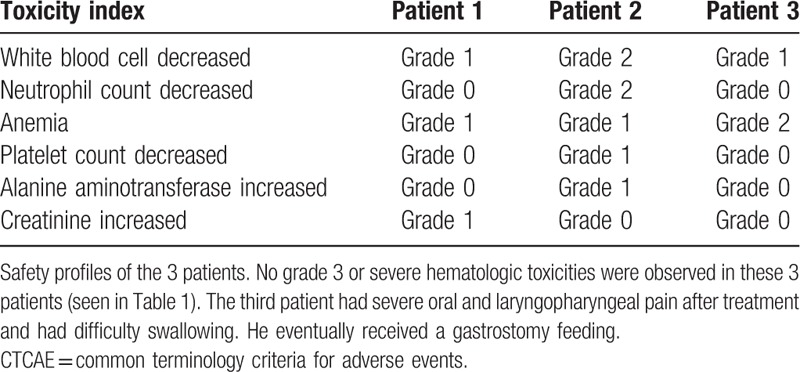
Hematologic toxicity in 3 patients during treatment according to CTCAE V 5.0.

### 3.2 Case 2

**Figure 2:**
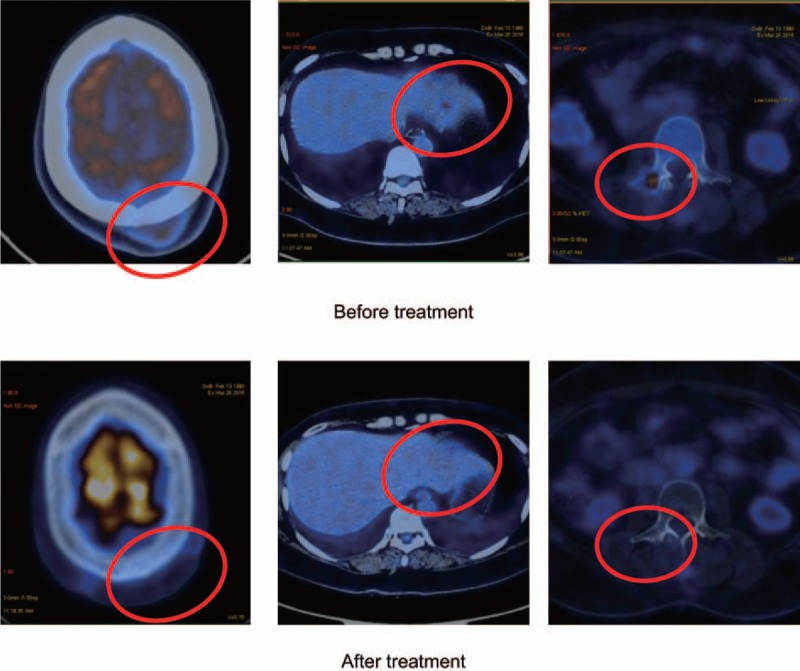
Imaging evaluation of treatment response for patient 2. Before treatment (in March 2016), lesions in the S2 regions of the liver and the lumbar spine L3 region were observed. After treatment (September 2016), no abnormality in the skull, liver, or L3 region were observed.

A 36-year-old female patient was first diagnosed with stage III T1N3M0 NPC in July 2014. She was treated with concurrent IMRT (68 Gy/30 Fr) and 2 cycles of nedaplatin (130 mg IV drip, d1, q28d) combined with 5-Fu (3.25 g civ120 h, q28d). At a follow-up visit in December 2015, lumps were seen in the left parieto-occipital region on MRI and PET/CT scans, and the plasma EBV DNA load measured 2.4 × 10^3^ copies/mL. In January 2016, biopsy analysis found pathological evidence that the intracranial, cranial, and scalp lumps were metastases from NPC. In January 2016, the patient was treated with radiation therapy to the left parieto-occipital region (36 Gy/12 F) concurrent with 5-Fu (200 mg/m^2^ civ240 h) chemotherapy. No abnormality was observed in the skull in PET/CT scan at a follow-up visit in March 2016; however, lesions in the S2 regions of the liver and the lumbar spine L3 region were observed (Fig. 2, before treatment).

The patient underwent 3 cycles of our proposed therapy with concurrent radiation therapy (12 Gy/12 F for liver metastases and 24 Gy/12 F for the clinical target volume [CTV] of bone metastases at levels L2–4) from April to August 2016. The patient showed CR as evaluated by RECIST 1.1 in September 2016 with no detectable plasma EBV DNA load and no abnormality in the skull, liver, or L3 region on PET/CT scan (Fig. 2, after treatment). Hematologic toxicity of the treatment is acceptable (Table 1).

### 3.3 Case 3

**Figure 3:**
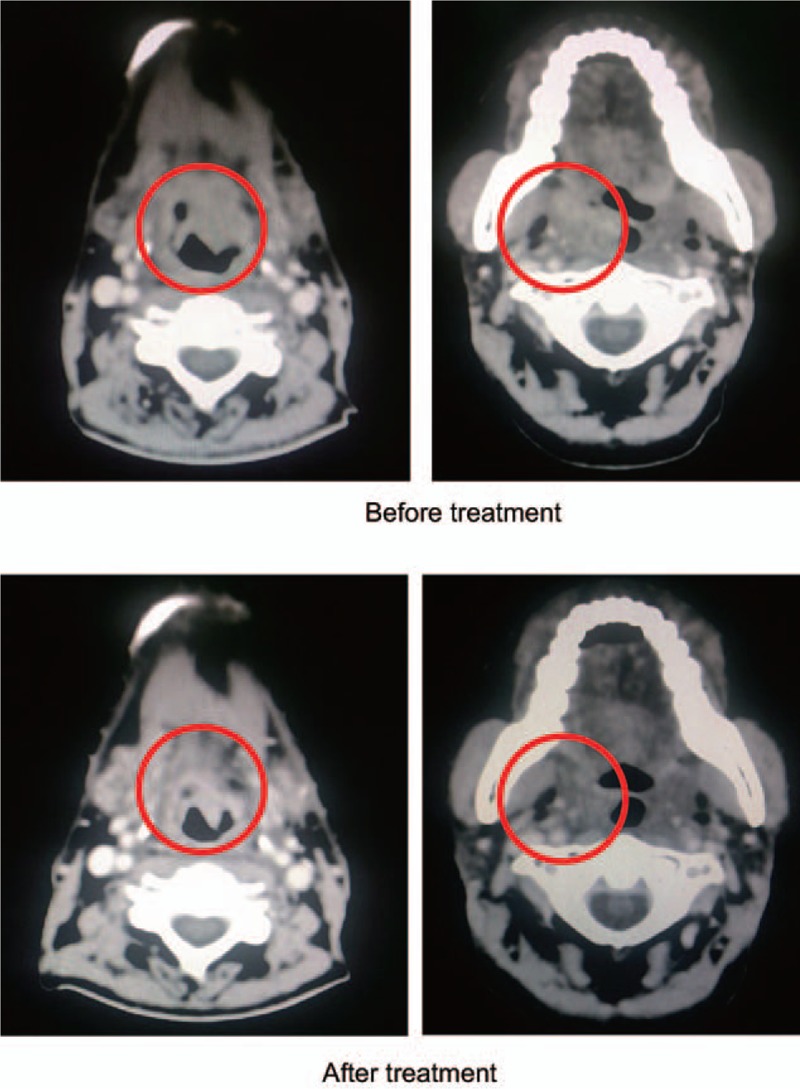
Imaging evaluation of treatment response for patient 3. Before treatment (January 2015), hypopharynx recurrence with cervical lymph node enlargement was observed. After treatment (April 2015), the tumors in the hypopharynx and neck regions showed partial responses.

A 46-year-old male patient was diagnosed with stage II, T2N1M0 NPC in 2011 in Fudan University Shanghai Cancer Center. From June to July 2011, he received radical radiation therapy and showed complete response in both nasopharynx and neck lymph nodes. In July 2013, he was diagnosed with a nasopharynx recurrence and was treated with 2 cycles of chemotherapy (docetaxel 120 mg and DDP 40 mg) and reirradiation of the recurrent site with dose 64 Gy/27 Fr. He refused to continue with chemotherapy until recurrence progression with dysphagia in Aril 2014. He received 1 cycle of chemotherapy (Nedaplatin 80 mg/m^2^ D1, D28, 5-Fu 200 mg/m^2^/d civ24 h d1-30) combined with concurrent radiotherapy to recurrence site (15 Gy/15 Fr, then shrink field boost with 21 Gy/7 Fr) from May to June 2014. Partial relief of dysphagia was achieved. He refused to continue with treatment again. In August 2014, he suffered aggravation of dysphagia and started treatment again. Oral chemotherapy (Tegafur) was administered, while it did not appear to work. Imaging showed hypopharynx recurrence with cervical lymph node enlargement in January 2015 (Fig. 3, before treatment).

From January to April 2015, the patient underwent 2 cycles of our proposed therapy. Radiation therapy was given (15 Gy/15 F and 4.5 Gy/6 F) to hypopharynx concurrently. The tumors in the hypopharynx and neck regions showed partial responses as evaluated by RECIST 1.1 (Fig. 3, after treatment). Hematologic toxicity of the treatment is acceptable (Table 1).

## 4 Discussion

Despite an optimistic prognosis for NPC, refractory NPC is associated with a significant mortality rate. The challenge in treating advanced NPC lies in the fact that some patients become refractory to treatment and intolerant to reirradiation or intensive chemotherapy.

The definition of refractory NPC is not consistent across studies. Zhang et al^[16]^ defined patients with refractory NPC as those who had recurrence tumors but cannot tolerate surgical removal or reirradiation and chemotherapy after adequate doses of radiotherapy. Gao et al,^[17]^ in contrast, defined NPC that did not respond to previous chemotherapy regimens as refractory. Our definition of refractory NPC included the following 3 categories: recurrence with radiation brain injury after radiotherapy, recurrence after the second or more courses of radiotherapy, and first-line treatment failure with multiple distant metastases (either synchronous or metachronous). Currently, there are no consistent guidelines for the treatment of refractory NPC as defined above, and the current study aims to explore possible treatment options for it.

In this case report, we presented 2 cases of refractory NPC that showed complete response and 1 case showed partial response to Endostar treatment combined with chemotherapy and radiation therapy. The EBV DNA load was undetectable in both patients who showed complete response and the local and distant tumors showed regression after treatment with Endostar.

Judah Folkman has proposed that the optimal effects of an antiangiogenic therapy require endothelial cells be exposed to steady blood levels of the agent, and at too high or too low a level, the agent will be ineffective.^[18]^ Endostatin injected intraperitoneally was found to be rapidly cleared within 2 hours in a mouse xenograft tumor model, although it remained stable and active in mini-osmotic pumps for at least 7 days. Continuous intraperitoneally administration of endostatin results in more effective tumor suppression compared with bolus administration in this model.^[19]^ A phase I study of pharmacokinetic in patients with advanced cancer^[20]^ showed that 4-week continuous intravenous infusion of recombinant human (rh)-endostatin was safe. Therefore, in our study, Endostar was administered continuously via intravenous infusion (2 mL/h for 30 days each cycle, for a total of 6 pumps [120 hours for each pumps] each cycle).

Continuous intravenous infusion of 5-Fu has been proposed by Lokich et al^[21]^ in 1981. They found that the treatment did not require interruption for up to 60 days at dose rates of 300 mg/m^2^/d or less.^[21]^ Fandi et al^[22]^ reported that treatment with low-dose continuous infusion 5-fluorouracil in recurrent and/or metastatic undifferentiated NPC was safe and effective. Administration of 5-Fu (300 mg/m^2^/d) for 6 weeks resulted in an objective response rate of 25% and mild toxicity.^[22]^ Therefore, a continuous intravenous infusion of 5-Fu at the dose of 200 mg/m^2^/d for 30 days in the treatment of refractory NPC was considered safe and effective.

Platinum-based chemotherapy is still the gold standard for NPC. However, patients with refractory NPC have poor tolerance to intensive chemotherapy regimen. Cisplatin is associated with toxicities in the gastrointestinal tract and the kidney. Compared with cisplatin, nedaplatin is associated with relatively mild adverse events with a similar antitumor effect in head-and-neck cancer.^[23,24]^ In a multicenter phase II study of recurrent and metastatic NPC after failure of cisplatin-based chemotherapy, Peng et al^[25]^ reported that oral capecitabine (1000 mg/m^2^ twice daily from days 1 to 14) combined with intravenous nedaplatin (80 mg/m^2^, day 1) offers a satisfactory clinical activity and an acceptable safety profile. We selected nedaplatin (80 mg/m^2^/d, iv drip on D1 and D28 each cycle) in our chemotherapy regimen based on the optimal safety and efficacy profile of nedaplatin.

Overall, in this study of 3 patients with refractory NPC, Endostar combined with chemotherapy and/or radiation therapy resulted in complete response in 2 patients and partial response in a third patient, as well as reduced serum EBV load. There results indicated that Endostar in combination with chemotherapy and radiation therapy could be an effective treatment of refractory NPC. Enrollment for patients is open for a large-scale clinical trial to assess the efficacy and safety of continuous infusion of Endostar in combination with chemotherapy and radiation therapy.

## Author contributions

**Supervision:** Yun-Fei xia.

**Writing – original draft:** Chen Chen, Song-Ran Liu, Shu Zhou, Xiao-Hui Li, Xiao-Hui Wang, Ya-Lan Tao, Hui Chang, Wen-Wen Zhang, Wen-Fei Li, Si-Lang Zhou.
